# Supplementary material for: Accuracy of long-term volunteer water monitoring data: A multiscale analysis from a statewide citizen science program
Source: PLoS One. 2020 Jan 29;15(1):e0227540. doi: 10.1371/journal.pone.0227540 (PMC6988940; doi:10.1371/journal.pone.0227540)
Supplement: Attachment — Submitted filename: Response to Reviewers_second response.docx [file pone.0227540.s002.docx]

Response to Reviewers

On page 8 of the manuscript, in parentheses, we wrote Table 3 when we meant Table 1. We have made this edit. We apologize for any inconvenience.

Response to Reviewers

PONE-D-19-25900
Accuracy of long-term volunteer water monitoring data: A multiscale analysis from a statewide citizen science program
PLOS ONE

Journal Requirements:

When submitting your revision, we need you to address these additional requirements.

1. **Please ensure that your manuscript meets PLOS ONE's style requirements, including those for file naming. The PLOS ONE style templates can be found at:**

[http://www.journals.plos.org/plosone/s/file?id=wjVg/PLOSOne_formatting_sample_main_body.pdf](https://nam04.safelinks.protection.outlook.com/?url=http%3A%2F%2Fwww.journals.plos.org%2Fplosone%2Fs%2Ffile%3Fid%3DwjVg%2FPLOSOne_formatting_sample_main_body.pdf&data=02%7C01%7CKelly.Albus%40unt.edu%7Cc5f90e2a175340b573a408d754186f98%7C70de199207c6480fa318a1afcba03983%7C0%7C0%7C637070336238492528&sdata=Co7N22nOtScNgcgLTBJNgaO2ZNn5U01oxv12lESo80g%3D&reserved=0) and [http://www.journals.plos.org/plosone/s/file?id=ba62/PLOSOne_formatting_sample_title_authors_affiliations.pdf](https://nam04.safelinks.protection.outlook.com/?url=http%3A%2F%2Fwww.journals.plos.org%2Fplosone%2Fs%2Ffile%3Fid%3Dba62%2FPLOSOne_formatting_sample_title_authors_affiliations.pdf&data=02%7C01%7CKelly.Albus%40unt.edu%7Cc5f90e2a175340b573a408d754186f98%7C70de199207c6480fa318a1afcba03983%7C0%7C0%7C637070336238502525&sdata=WF8NQx0Kft3HZhQwDwlUIiIrxjr7%2FiTcl3CTh40SuCc%3D&reserved=0)

The authors have reviewed the manuscript and associated figure files and to the best of our knowledge they meet all of PLOS ONE’S style and file naming requirements.

2. **In your Methods section, please include additional information about your dataset and ensure that you have included a statement specifying whether the collection method complied with the terms and conditions for the websites from which you have collected data.**

The authors appreciate the editors and reviewers’ comments on this point and agree that the language for the data accessibility was unclear in the original submission. To clarify, all raw data used for analyses is publicly accessible by state regulations, both professional and volunteer, and we have added more information about this in the references as well as an additional sentence on p. 5 of the manuscript. There is also more detailed information about data availability in responses to individual reviewer comments below.

Also to clarify regarding any restrictions on the data: all data used was accessible online via database portals housed by third parties, and all associated data sharing policies are included on the website, and all links are now provided in the references. Authors have added an additional database link in the manuscript and in references [32], which includes an updated version of the volunteer online data portal with an increased data sharing and policy description.

3. **We note that Figure [3] in your submission contains a map image which may be copyrighted. All PLOS content is published under the Creative Commons Attribution License (CC BY 4.0), which means that the manuscript, images, and Supporting Information files will be freely available online, and any third party is permitted to access, download, copy, distribute, and use these materials in any way, even commercially, with proper attribution. For these reasons, we cannot publish previously copyrighted maps or satellite images created using proprietary data, such as Google software (Google Maps, Street View, and Earth). For more information, see our copyright guidelines:** [**http://journals.plos.org/plosone/s/licenses-and-copyright**](https://nam04.safelinks.protection.outlook.com/?url=http%3A%2F%2Fjournals.plos.org%2Fplosone%2Fs%2Flicenses-and-copyright&data=02%7C01%7CKelly.Albus%40unt.edu%7Cc5f90e2a175340b573a408d754186f98%7C70de199207c6480fa318a1afcba03983%7C0%7C0%7C637070336238502525&sdata=zVismoHPMRugO8nK7vIpXXijFVbztQGgUbG78QqczVg%3D&reserved=0)**.**

**We require you to either (1) present written permission from the copyright holder to publish these figures specifically under the CC BY 4.0 license, or (2) remove the figures from your submission:**

We appreciate the editor’s attention to detail and comprehensive suggestions, and also fully support Plos One’s commitment to making scientific knowledge freely available. The authors have chosen to remove the map (Figure 3) from the final manuscript as they feel it does not provide a significant contribution to the result and would require unnecessary time, manuscript space and references.

4. **Please upload a copy of Supporting Information Figures and Tables which you refer to in your text on page 28.**

Thank you for your suggestion, and our apologies for the confusion - there are no supporting figures to accompany the text, so this page was removed from the manuscript.

Additional Editor Comments (if provided):

Dear Dr. Thompson,
Thank you for considering PLOS ONE for your manuscript submission. It has been forwarded to reviewers for their consideration, and the reviewers recommend reconsideration of your paper following major revision. I invite you to resubmit your manuscript after addressing all reviewer comments. When resubmitting your manuscript, please carefully consider all issues mentioned in the reviewers' comments, outline every change made point by point, and provide suitable rebuttals for any comments not addressed.

I look forward to receiving your revised manuscript as soon as possible.

Kind Regards,
Saifur Rahaman

[Note: HTML markup is below. Please do not edit.]

Reviewers' comments:

Reviewer's Responses to Questions

**Comments to the Author**

1. Is the manuscript technically sound, and do the data support the conclusions?

The manuscript must describe a technically sound piece of scientific research with data that supports the conclusions. Experiments must have been conducted rigorously, with appropriate controls, replication, and sample sizes. The conclusions must be drawn appropriately based on the data presented.

Reviewer #1: Partly

Reviewer #2: Partly

Reviewer #3: Yes

The authors address these below as responses to individual Reviewer comments.

2. Has the statistical analysis been performed appropriately and rigorously?

Reviewer #1: Yes

Reviewer #2: Yes

Reviewer #3: Yes

 The authors address these below as responses to individual Reviewer comments.

1. Have the authors made all data underlying the findings in their manuscript fully available?

   The [PLOS Data policy](https://nam04.safelinks.protection.outlook.com/?url=http%3A%2F%2Fwww.plosone.org%2Fstatic%2Fpolicies.action%23sharing&data=02%7C01%7CKelly.Albus%40unt.edu%7Cc796d23360d743396ad308d753d1a02a%7C70de199207c6480fa318a1afcba03983%7C0%7C0%7C637070032110392098&sdata=XCB0vI2DYCBBHq7dEb%2Bv%2B1sAE1Q0KgaXuJKVptW1T9I%3D&reserved=0) requires authors to make all data underlying the findings described in their manuscript fully available without restriction, with rare exception (please refer to the Data Availability Statement in the manuscript PDF file). The data should be provided as part of the manuscript or its supporting information, or deposited to a public repository. For example, in addition to summary statistics, the data points behind means, medians and variance measures should be available. If there are restrictions on publicly sharing data—e.g. participant privacy or use of data from a third party—those must be specified.

   Reviewer #1: No

   Reviewer #2: No

   Reviewer #3: Yes

Author response: The language for the data accessibility was unclear, and we appreciate the editor’s and reviewers’ comments on this point. All raw data used for analyses is publicly accessible by state regulations, both professional and volunteer, and we have clarified this with additional information in the references section as well as an additional sentence on p. 5 of the manuscript. There is also additional information about data availability in responses to individual reviewer comments below.

Also, to clarify regarding any restrictions on the data: all data used was accessible online via database portals housed by third parties, in which all of their rights, etc. are included on the website links provided in the references. Authors have added a database link in the manuscript and in references [32], which includes an updated version of the volunteer online data portal with an increased data sharing and policy description.

4. Is the manuscript presented in an intelligible fashion and written in standard English?

PLOS ONE does not copyedit accepted manuscripts, so the language in submitted articles must be clear, correct, and unambiguous. Any typographical or grammatical errors should be corrected at revision, so please note any specific errors here.

Reviewer #1: No

Reviewer #2: Yes

Reviewer #3: Yes

 The authors address these below as responses to individual Reviewer comments.

Review Comments to the Author

**----------------------------------------------------------------------------------------------------------------------------**

**Reviewer #1: General Comments:
The study focuses on a very important evaluation of accuracy of the volunteer water quality monitoring program. It is an interesting study. The authors wanted to emphasize the importance of accuracy in the long term. However, water quality in natural water bodies vary widely even throughout the year. Long term accuracy doesn’t really guarantee short term suitability. During the time of pollution, it is not clear as to how the long term accuracy will ensure emergency response. The authors conducted a five day study to evaluate the short term implications. But, 21 samples on 10 different locations are good. However, two samples in each locations are not that high.**

The authors are glad Reviewer 1 found the study interesting and would like to thank them for their time and attention to detail in reviewing our manuscript. We also agree with Reviewer 1 that the variability of long-term datasets will be high due to natural variability in ecosystems over time, and this is mentioned in the Discussion section of the manuscript when referring to the natural variations within the datasets, especially at a statewide scale (p. 13). We hope that by utilizing this highly variable, existing volunteer data to perform a post-hoc, multi-scaled analysis on a large-scale dataset, without experimental controls in place, that we can provide a reproducible framework for future analyses of other such existing datasets. The agreement between volunteers and professionals would then be in spite of the multiple sampling techniques conducted by numerous volunteer and professional entities over the years and a large geographic area with diverse ecosystems.

Reviewer 1 makes a good point that short-term suitability, especially in regards to pollution events and emergency response, is not necessarily aided by these analyses of long-term datasets. The focus of this study was on the benefits of long-term data specifically, which are useful for management concerns such as baseline data, trend analysis, or assessing change over time in response to population growth or as evidence for land-use change impacts (p.3). Long-term accuracy was evaluated by conducting an analysis of the data in its entirety, which implies that any part of the dataset, taking separately, is of high enough quality to be used as needed, either for long-term studies or for a short-term response. However, the short-term uses, especially in regards to pollution or emergency recovery, are excellent areas for future research, and thanks to the reviewers suggestion, we have added the following sentence to the manuscript “Future research with existing, long-term volunteer data could further evaluate their uses when combined with professional resources in pollution remediation or emergency response efforts (p.15)”.

Reviewer 1 also brings up a valid concern about the amount of sample data from each sample site for the experimental dataset (42 samples, 21 volunteers and 21 professionals collected from 10 different sites). If taken alone, we recognize that 2 volunteer and 2 professional samples from each site would not be a statistically sound result. This is why the experimental dataset was only run as a group, or pooled, result for which all ANOVAs apply only to the entire 42 sample dataset. Again, we recognize that this is not as much data as could be hoped for, but we believe that in conjunction with the larger, existing dataset (also from the same sources) it provides an important piece of the puzzle.

Specific Comments:
**1. The quality of the figures are not good. Needs improvement.**

Reviewer 1 is entirely correct on this point, and the author has improved the quality of figures.

2. **Different headings used in different sections are confusing. For example “Volunteer and professional stream water quality monitoring” and “Statewide volunteer vs. professional data”. I couldn’t understand the differences between the two headings and the content inside them. Then you have another section with “City of Denton volunteer vs. professional data”. Please reconsider the headings. I think a generic name reflecting the big idea would be preferable.**

We recognize Reviewer 1’s confusion on this point and appreciate the suggestion to have headings that better reflect the overall idea. Authors have removed the ““Volunteer and professional stream water quality monitoring” heading, changed the “Statewide volunteer vs. professional data” heading to a more generic “Data collection and analysis”, and also removed the “City of Denton volunteer vs. professional data” heading. We agree that this makes for a clearer manuscript with less confusion about the sections and would like to thank the reviewer for their input.

3. **In the section Statewide volunteer vs. professional data, statistical analysis is discussed. The authors mentioned about data collection and the years of data collection. However, the actual number of data used in the analysis is not clear. For example, 15 samples in 15 years are different to 15 samples in one year.**

We believe Reviewer 1 is referring to the statewide dataset specifically, although this is one of three research questions, the other two conducted the same way. For all analyses, the raw data is available for more detail. For the statewide portion, however, we ran a pooled analysis by year, as explained in the Methods: “Samples were selected for analyses when samples were collected: 1) at a station for which both TST and TCEQ had a corresponding station (i.e., one with the same Station ID or located within 60 meters of the same segment), and 2) during a year in which both TST and TCEQ sampled stream segments two or more times (station year). Due to unknown sampling techniques, variable sampling dates and times, and the large geographic area covered, these data were not analyzed as paired samples for the statewide dataset. Instead, the data were pooled and analyzed by year.” (p. 6) This means that all of the samples collected in that year (minimum of 2 volunteer samples and 2 professional samples for that year to be included, and no more than 12-13 samples per year per source as the most frequent sampling was monthly) were pooled together to run for that year, at that station, for that parameter. This was done because non-paired samples can’t be compared directly (different times of the year, different conditions), so the analysis of variance was applied only to the combined results, to determine if the variation between professionals and volunteers was significant across all values.

However, Reviewer 1 is justified in being concerned about the number of data points included in the analysis for statistical accuracy, and the authors would like to clarify that each year was used only AFTER determining that the data for that year was normally distributed. Thanks to Reviewer 1’s suggestion, we have added a sentence to the manuscript to explain this more clearly (“Instead, the data were pooled and analyzed by year, after determining that data were normally distributed for that year.” (p.6))

**4. The data visible in the Table 2, are quite few. Analysis based on these data are difficult to conclude.**

The authors would like to clarify that the data included in Table 2 (and also in Table 1) were the final ANOVA results, not the actual data used in the analyses. The data used for analysis were more extensive, and described in paragraph above Table 2: “a total of 159 volunteer and professional samples from 2009-2016, resulting in 70 analyses (ANOVAs for DO and conductivity and KST for pH), each one representing an analysis of variance between the volunteers and professionals at one station for one year and for one of the parameters (DO, pH or conductivity) (Table 2). Results included volunteer and professional data comparisons from 6 stations for a total of 24 separate station years of sampling data.” (p.10)

**5. Experiments done by Group M were mentioned in the discussion. However, how many groups were there? There were not enough details shown.**

Reviewer 1 makes a good point that the language for this was ambiguous and that more detail is needed here. The only group studied was Texas Stream Team; this section was a disclaimer that the majority of data from the City of Denton volunteer dataset had been collected by one set of 8-10 TST volunteers that have sampled together at those locations for many years consecutively, and therefore could influence the results. In response to Reviewer 1’s comment, the authors added additional language to clarify this in the “Dedicated volunteer bias” section: “The majority of the 2009-2016 City of Denton volunteer data were collected by a small number of dedicated TST volunteers that have been sampling as a group since the city began its partnership with TST. Four out of six stations analyzed and 63 out of the total 69 analyses were all sampled by this same group of volunteers, termed for this study as Group M, which has been sampling together consistently for 10+ years.” (p. 13-14)

**6. Where there were sections identifying the bias in the water quality monitoring, the accuracy can be questioned. This should be mentioned in the conclusion as well.**

Again, the authors would like to thank Reviewer 1 for their insight and suggestion and agree this is a good idea. The following sentence has been added to the conclusion, “Increased controls on multi-scale analyses identified a systematic bias in DO measurements and a pattern in pH data between volunteer and professional data which were consistent enough to be corrected for in further analysis.” (p.15)

**------------------------------------------------------------------------------------------------**

**Reviewer #2:**

**The manuscript presents a comparative study between professional (state-wide) water monitoring results and the ones from volunteer groups. The paper first has focused on a state-scale and then narrowed down the comparison at the city scale. I have the following comments for the authors to consider:**

**•The reviewer is still unsure about the main take away from this comparative study. Do authors want to advocate on sampling being done by volunteer group? And if so, upgrading their sampling methods and equipment?** **Is this advocated as part of a larger scale transition to a “social innovation” paradigm with reduced role and responsibility for the Governments? In that case who can be held accountable in case of monitoring errors and health-related consequences? The reviewer was in search of a wider motivation for this study.**

We would like to thank Reviewer 2 for their time in reviewing our manuscript, and for their thoughtful reflection regarding wider motivations and the main message behind the study. To provide context, this study is not advocating for specific action in regards to volunteer monitoring protocol, equipment, or increased responsibility as compared to Government entities. This study is only referencing the data that is or has been collected by volunteer monitoring groups that already exist. Many of these volunteer monitoring programs were created in part by the Government agency responsible for maintaining environmental standards. In this study, the TST program was initiated by the Texas Commission for Environmental Quality (TCEQ) for multiple reasons, including community outreach and to meet increased monitoring needs. The volunteer programs aren’t designed to replace or reduce governmental involvement; Reviewer 2 is entirely correct that monitoring errors and health-related consequences are not within their role. Instead, volunteer monitoring programs are serving as supplemental to regulatory officials, aiding with community education and involvement, as well as providing additional data.

The motivations for the results of this study are primarily to help inform pathways for volunteer data to be used alongside professional data in expanded capacities, areas where it may be needed most. Although citizen science has been an integral part of water quality monitoring and management for decades, the long-term, quality-assured datasets these programs have generated are often underutilized or undervalued simply because volunteers generated them. This study is hoping to show that volunteer data can be high quality data, especially if collected under a quality assurance plan.

However, Reviewer 2 makes an excellent point that these motivations are not made clear enough in the manuscript, especially in the beginning to help the reader frame the results as they move forward. The authors have added the following sentence to the Introduction section on page 4, “The results from this study can provide a more comprehensive understanding of the variations between volunteer and professional data over a volunteer monitoring program’s entire history.”

**•What is the importance of looking into these differences between the two groups of samples in “long-term”? Was the main purpose to create a larger data set? I believe that the authors could have tried to divide data to a number clusters based on sampling methods equipment characteristics and then to only compare the two groups cluster-by-cluster.**

Although Reviewer 2 brings up a valid research design for a further study, we believe that there is merit to these results as they stand. Long-term volunteer data that already exists could be very useful for predicting trends and showing changes over time if it was used alongside professional data. In the Introduction, we reference how long-term datasets are especially useful for providing baseline information on streams, identifying target areas for further sampling, and providing evidence for land-use change impacts [1, 20, 21, 22]. (p.3)

The purpose of utilizing the volunteer data in addition to professional data is not just to create a larger dataset, but one that is more complete. Volunteers often collected at different times and at different sites than professionals, so this is unique data to be included. More comprehensive data could lead to more efficient water quality management, and also better inform trend analysis and predictive models.

Reviewer 2’s suggestion of basing analyses on sampling methods and equipment certainly has merit. However the sampling methods are considered unknown for this study. These specifics were omitted by design as we believed the results (recorded measurements of DO, pH, and conductivity) should be comparable between professionals and volunteers independent of the specific protocols used, as long as both were operating under a QAPP based on the same EPA-approved protocols. [24, 32]. This was also done to encourage other researchers and management professionals to consider a similar analysis of their own existing data, even if they can’t obtain all of the associated details on sampling methods and equipment.

**•I suggest that the authors add a table summarizing the sampling characteristics in each monitoring group over the years, and if there has been any update during this time on sampling methods and equipment.**

This is a good suggestion and would be a great visual for future studies. However, as mentioned in the previous comment, the sampling methods and equipment are purposefully considered unknown for this study. One goal of this study and also the reasoning behind using existing data was to show that volunteer and professional datasets can be comparable even though the sampling methods and equipment are variables that cannot be controlled for after the fact. By not controlling for these, and instead treating sampling methods and equipment as an unknown, we can indicate that quality assurance protocols lead to good agreement between volunteers and professionals over time, regardless of inherent variation within protocols and equipment. This was done to show that this data can still be used without having to know all of those variables or correct for them. This is the takeaway for managers, etc. – you can use this data without knowing everything about how it was collected, because it was under a QAPP. We believe this more approachable method has the greatest capacity to impact future water quality management decisions.

**•It was not clear why the focus of the comparison was on DO, pH, and conductivity? What about BOD, Nitrate, phosphorous, and bacteria?**

Reviewer 2 brings up a good point that this was not fully clarified in the manuscript, although there was a clearer reason for it. Thank you for the suggestion; on pg. 5, we added the following sentence, “We compared three water quality parameters: dissolved oxygen (DO), pH and conductivity as these parameters are the most commonly assessed by both volunteers and professionals and for which there was sufficient sampling data for comparative analysis.”

**•The removal of bias/error from DO samples from Volunteers: How did the authors reach to a conclusion to add 2 mg/L to all samples? How did the authors arrive at this number?**

The raw data was visually assessed and it was apparent that the volunteer and professional values were off by only a few (1-3) mg/L across the entirety of the dataset. By also visually assessing the boxplot, we tried multiple mg/L values (1, 1.5, 2, 2.5, 3) and determined that by adding 2 mg/L and it corrected the bias (removing any significant difference between the two sources and could be visualized in the corrected boxplot).

**•Despite an emphasis expressed at the beginning of the article, not much of analysis was conducted on “conductivity” criteria.**

We believe Reviewer 2 is responding to the additional figures (Fig 2a and 2b for box plots of DO; Fig 4 and Fig 5 for empirical distribution for pH) which were included only for DO and pH in the two local (City of Denton) analyses and we can see how this could be misleading. To clarify, the same analyses were conducted for DO, pH and conductivity equally. Conductivity box plot and empirical distribution results did not provide additional information relevant to the study; the plots simply showed no significant variation in the conductivity measured between volunteers and professionals which was related in the text rather than as additional visuals. However, the DO and pH did show significant variation, and also the visuals themselves revealed additional information about the data distribution so they were included in the manuscript. Reviewer 2 is correct that there are fewer visuals for conductivity, which could easily make it appear as though conductivity had fewer statistical analyses conducted, but this was not the case.

**•Minor comments:**

**- There were no page numbers.**

Great point, this makes it more difficult to reference. Author added page numbers to manuscript.

**- Figure sizes shall be reduced.**

Agreed, and authors have increased quality of figures.

**------------------------------------------------------------------------------------------------**

**Reviewer #3:**

**This study assesses the reliability of large-scale volunteer water quality data by comparing it corresponding professionally collected data set. This is a well-written manuscript with systematic presentation and interpretation of data that can be followed quite well. I have a couple minor comments.**

**DO bias adjustment was not highlighted in the Abstract.**

We thank Reviewer 3 for their thoughtful review of this manuscript and their positive feedback and constructive comments. This is a good point; referencing the DO bias adjustment would make the abstract more complete. We have added language to the abstract (p.2) as follows: “Data from a single partner city, with increased controls on sampling times and locations and correction of a systematic bias in DO, confirmed this by showing an even greater agreement of 91% overall from 2009 -2017 (DO = 91%, pH = 83%, conductivity = 100%).”

**Please elaborate a little more (paragraph after Table 2) “By increasing analytical controls to …….. relative accuracy of volunteer data increased”.**

The authors appreciate that Reviewer 3 pinpoints an area where the language is ambiguous for an important point of the study and needs further clarification. It was not clear what was meant behind the increased controls on the local datasets, which differed from the statewide datasets by including only professional and volunteer samples that were paired to within 5 days of each other and at exact same sites, rather than only within the same year and from a site that was within 60 m of the other. On page 11, the following sentence was reworked to clarify this: “Unlike at the statewide scale, the local analysis included only volunteer and professional samples that were paired within a five-day range of each other and at the same monitoring stations. By increasing analytical controls to include only volunteer and professional data that were more directly comparable, the relative accuracy of volunteer data increased as well.”

6. PLOS authors have the option to publish the peer review history of their article ([what does this mean?](https://nam04.safelinks.protection.outlook.com/?url=https%3A%2F%2Fjournals.plos.org%2Fplosone%2Fs%2Feditorial-and-peer-review-process%23loc-peer-review-history&data=02%7C01%7CKelly.Albus%40unt.edu%7Cc796d23360d743396ad308d753d1a02a%7C70de199207c6480fa318a1afcba03983%7C0%7C0%7C637070032110392098&sdata=dBfIjEo9uIEYysFq7hz7oDKAUzNwT2zwWRvtGBqbFck%3D&reserved=0)). If published, this will include your full peer review and any attached files.

If you choose “no”, your identity will remain anonymous but your review may still be made public.

**Do you want your identity to be public for this peer review?** For information about this choice, including consent withdrawal, please see our [Privacy Policy](https://nam04.safelinks.protection.outlook.com/?url=https%3A%2F%2Fwww.plos.org%2Fprivacy-policy&data=02%7C01%7CKelly.Albus%40unt.edu%7Cc796d23360d743396ad308d753d1a02a%7C70de199207c6480fa318a1afcba03983%7C0%7C0%7C637070032110402092&sdata=0QZ7SLwBTraX%2FiS3YHEodhXRojMSxvlSHSBGeLUj%2B3s%3D&reserved=0).

Reviewer #1: No

Reviewer #2: No

Reviewer #3: No


[NOTE: If reviewer comments were submitted as an attachment file, they will be attached to this email and accessible via the submission site. Please log into your account, locate the manuscript record, and check for the action link "View Attachments". If this link does not appear, there are no attachment files to be viewed.]

While revising your submission, please upload your figure files to the Preflight Analysis and Conversion Engine (PACE) digital diagnostic tool, [https://pacev2.apexcovantage.com/](https://nam04.safelinks.protection.outlook.com/?url=https%3A%2F%2Fpacev2.apexcovantage.com%2F&data=02%7C01%7CKelly.Albus%40unt.edu%7Cc796d23360d743396ad308d753d1a02a%7C70de199207c6480fa318a1afcba03983%7C0%7C0%7C637070032110402092&sdata=%2FlG6wVzEckpa4Rxqf6x39Mt9%2BWb5pUnY4wGpimZpg68%3D&reserved=0). PACE helps ensure that figures meet PLOS requirements. To use PACE, you must first register as a user. Registration is free. Then, login and navigate to the UPLOAD tab, where you will find detailed instructions on how to use the tool. If you encounter any issues or have any questions when using PACE, please email us at [figures@plos.org](mailto:figures@plos.org). Please note that Supporting Information files do not need this step.

*In compliance with data protection regulations, you may request that we remove your personal registration details at any time.* [*(Remove my information/details)*](https://nam04.safelinks.protection.outlook.com/?url=https%3A%2F%2Fwww.editorialmanager.com%2Fpone%2Flogin.asp%3Fa%3Dr&data=02%7C01%7CKelly.Albus%40unt.edu%7Cc796d23360d743396ad308d753d1a02a%7C70de199207c6480fa318a1afcba03983%7C0%7C0%7C637070032110402092&sdata=c3NDjkz8JCCYSUiBaKAsacVWMmfXQS18Gsv5QTetJXI%3D&reserved=0)*. Please contact the publication office if you have any questions.*
